# Outcomes of Ovulation Induction Aimed to Pregnancy in Eight Hypopituitarism Patients

**DOI:** 10.7759/cureus.58819

**Published:** 2024-04-23

**Authors:** Yu Horibe, Akira Nakabayashi, Shuko Murata, Tomomi Hashimoto, Tsutomu Tabata

**Affiliations:** 1 Gynecology, Tokyo Women's Medical University, Tokyo, JPN; 2 Obstetrics and Gynecology, Tokyo Women's Medical University, Tokyo, JPN

**Keywords:** women fertility, controlled ovarian stimulation, hypopituitarism, assisted reproductive technology, infertility treatment

## Abstract

Background: Female sex hormones work in concert. Gonadotropin-releasing hormone and ovulation-inducing agents are required in female patients with infertility owing to hormone dysregulation. Although drug-induced follicular development can be expected in patients with endogenous female hormone deficiency, data are lacking on the protocols and drugs used.

Methods: We retrospectively examined the success rates of ovulation induction, assisted reproductive technology, and pregnancy outcomes in 66 cycles of eight patients with pituitary insufficiency at our hospital.

Results: Ovulation occurred in 75.4% (49/66); 82.6% (38/46) of patients <40 years and 57.9% (11/19) of patients ≥40 years of age. Five of the eight patients became pregnant, and three delivered babies. The fertilization rate was 78% with in vitro fertilization, and the recombinant follicle-stimulating hormone usage was 3,717.1 ± 1,528.9 International Unit in hypopituitarism patients.

Conclusion: Hypopituitarism patients can achieve ovulation, pregnancy, and delivery after optimal gonadotropin administration. Further studies are needed to determine the effects of gonadotropins on other pituitary hormones, such as growth hormones.

## Introduction

Female sex hormones, including gonadotropin-releasing hormone (GnRH), secreted by the hypothalamus, function in concert with the hypothalamus, pituitary gland, ovaries, and uterus [1]. In assisted reproductive technology (ART), regulated ovarian stimulation with female hormones effectively improves the reproductive success rate by acting through these mechanisms. However, ART is also needed for some female infertility patients whose pituitary gland dysfunction has disrupted the regulation of female hormones [2]. Although follicle development can be induced by administering drugs to patients with endogenous female hormone deficiencies, the protocols and drugs used have not been studied. A few studies have reported the usefulness of hormone replacement in female infertility, other than the hormones secreted by the pituitary gland. We retrospectively reviewed our experience with ART in patients with pituitary insufficiency, including treatment details and pregnancy outcomes.

## Materials and methods

We included patients who had undergone ovarian stimulation with gonadotropins between 2017 and 2022 at our hospital with hypopituitarism and luteinizing hormone (LH) and follicle-stimulating hormone (FSH) levels below the detection sensitivity.

The resultant cases were extracted for a total of eight patients: one with idiopathic hypopituitarism, one with Sheehan syndrome, four with pituitary insufficiency after surgery for pituitary tumor, one with cavernous hemangioma, and one with anorexia nervosa, respectively. The details are described in Table 1.

**Table 1:** Diagnosis and background of patients with pituitary hormonal disorders ACTH: adrenocorticotropic hormone

No	Age at treatment (years)	Cause	Gonadotropin	Growth hormone	Thyroid stimulating hormone	ACTH	Vasopressin
			Depends on menstrual cycle	0.13-9.88(ng/mL)	0.35-4.94 (μIU/mL)	7.2-63.3 (pg/mL)	<2.8 (pg/mL)
1	39–42	Idiopathic	FSH 3.4, LH 1.0	0.34	2.3	30.4	NA
2	43–44	Sheehan syndrome	FSH 0.6, LH<0.2	0.07	0.023	<2.0	NA
3	33–37	Postoperative for pituitary tumor	FSH 1.4, LH<0.2	0.06	0.009	41.6	0.2
4	30–31	Postoperative for pituitary tumor	FSH 3.0, LH0.8	0.12	0.256	35.2	0.5
5	29–31	Cavernous hemangioma	FSH<0.1, LH<0.2	1.05	<0.005	12.2	1.7
6	36–37	Postoperative for pituitary tumor	FSH 3.6, LH 1.3	0.19	0.015	<2.0	0.5
7	39–40	Postoperative for pituitary tumor	FSH 0.4, LH<0.2	NA	0.01	<2.0	0.8
8	30	Anorexia nervosa	FSH<0.1, LH<0.2	NA	0.952	NA	NA

A total of 66 cycles were administered to eight patients. The following points describe each case. (1) A patient with idiopathic amenorrhea who had not been examined by an endocrinologist and had been treated with Kaufman therapy since the age of 20; (2) A patient with Sheehan’s syndrome due to heavy bleeding during delivery, resulting in thyroid dysfunction, adrenal insufficiency, growth hormone (GH) dysfunction, and gonadal dysfunction, who were receiving hydrocortisone 10 mg and levothyroxine sodium hydrate 125 μg/day; (3) A patient who underwent Hardy surgery for a pituitary medullary aneurysm and developed thyroid dysfunction, GH dysfunction, gonadal dysfunction, and central enuresis, and was treated with desmopressin acetate hydrate 120 μg and levothyroxine sodium hydrate 125 μg/day; (4) A case in which the patient was treated with levothyroxine sodium hydrate 100 μg/day after pituitary surgery for Latke’s cyst; (5) A patient with rheumatoid arthritis, pituitary insufficiency owing to multiple cavernous hemangiomas, and thyroid dysfunction using levothyroxine sodium hydrate 100 μg/day; (6) A patient who developed panhypopituitarism owing to pituitary surgery and received hydrocortisone 15 mg, levothyroxine sodium hydrate 50 μg, desmopressin acetate hydrate 60 μg, and somatropin 0.15-0.25 mg/day; (7) A patient who developed hypopituitarism after pituitary adenoma surgery and was receiving hydrocortisone 10 mg, levothyroxine sodium hydrate 62.5 μg, and somatropin 0.3 mg/day; (8) A patient who was receiving Kaufman therapy for anorexia nervosa.

First, we determined the success rate of ovulation induction. The success rate of ovulation was studied in two groups: <40 and ≥40 years of age. Second, the amount of gonadotropin used was compared between the ovulatory and non-ovulatory groups. Third, the amount of recombinant FSH (rFSH) used in in vitro fertilization (IVF) was examined in the control group which included randomly selected 20 non-pituitary dysfunction patients. Fourth, the follicle development, number of eggs obtained, and fertilization rate in IVF are summarized. Fifth, we summarized the delivery outcomes of the pregnancies. The Student’s t-test was used for statistical analysis. We obtained informed consent from all patients.

## Results

Ovulation

The overall ovulation rate was 49 of 66 cycles (75.4%): 35 of 49 cycles in the rFSH group and 14 of 16 cycles (87.5%) in the human menopausal gonadotrophin (hMG) group. One patient was administered a combination of clomiphenes and one patient in the hMG group developed ovarian hyperstimulation syndrome. In terms of follicle development, cases with good ovulation were case 5 with four cycles, case 6 with three cycles, and case 7 with 11 cycles, which was 100%. The patients with poor ovulation were case 1 (10 of 16 cycles, 62.5%), case 3 (five of eight cycles, 62.5%), and case 4 (six of 11 cycles, 54.5%).

When divided by age, 38 of 46 cycles (82.6%) developed in patients <40 years, and 11 of 19 cycles (57.9%) developed in patients ≥40 years of age. The lack of follicular development was particularly noticeable in cycles after 40 years of age. In addition, ovulation was observed in five of the eight cases after the first treatment. In cases 2 and 4, ovulation was not observed after the initial stimulation; however, subsequent ovulation was observed after adjustment for gonadotropin preparation based on the results of the initial stimulation.

rFSH dosage

The rFSH group aimed for single follicle development, and 19 of the 27 patients ovulated (70.4%). The ovulation group used 3,017.1 ± 1,183.1 International Units (IU) of rFSH, while the non-ovulation group used 2,196.9 ± 800.5 IU of rFSH. The two groups had no statistically significant difference (p = 0.09).

rFSH and ART

We examined the patients in the rFSH group using IVF. Follicular development was observed in 16 of the 22 cycles (72.3%) and 3,717.1 ± 1,528.9 IU of rFSH were used. In patients undergoing IVF without complications, the dose of rFSH used in 20 cycles of the randomly selected rFSH monotherapy group was 1,551 ± 404 IU, a statistically significant difference (8.4 × 10-60, [confidence interval 1.947-1.986]) (p<0.001).

Results of IVF

Controlled ovarian stimulation (COS) was administered during a total of 29 cycles. Table 2 lists the egg retrieval cycle, in which follicle development was observed, the number of follicles developed, the number of oocytes obtained, the fertilization rate, and the number of embryos available for transfer (Table 2). Statistical analyses were not performed because of the small sample size. The overall fertilization rate was high (78%) when oocytes were acquired. A sufficient number of oocytes were obtained in cases 4-6.

**Table 2:** Summary of egg retrieval in ovarian stimulation cycles in in vitro fertilization for each patient

No.	Cycles	Egg glow cycles	Egg glow	Egg retrieval	Fertilization	Enable ET
1	16	10	1 / 6 cycles; 2 / 2 cycles; 3 / 1 cycle	1 / 3 cycles; 2 / 3 cycles	7 / 9 ova	5 ova
2	2	2	1 / 1 cycle; 3 / 1 cycle	0 / 1 cycle; 2 / 1 cycle	1 / 2 ova	1 ovum
3	0	0	0	0	0	0
4	1	1	18	8	7 / 8 ova	7 ova
5	1	1	11	10	5 / 10 ova	5 ova
6	2	2	7 / 1 cycle; 13 / 1 cycle	7 / 1 cycle; 9 / 1 cycle	6 / 16 ova	6 ova
7	0	0	0	0	0	0
8	0	0	0	0	0	0

Pregnancy

Five of the eight cases resulted in pregnancy. Seven pregnancies occurred in all cycles: two by timing therapy, two by artificial insemination with the husband's semen, and three by frozen embryo transfer during hormone replacement cycles. Three cases resulted in the delivery of babies: two by timed therapy and one by frozen embryo transfer.

In case 3, the patient had a history of myocarditis and atypical angina pectoris; therefore, an elective cesarean section was performed at 37 weeks and three days for maternal indication. The baby weighed 2,712 g and was admitted to the NICU with an Apgar score of 1 point at 1 minute and 3 points at 5 min because of fetal distress. There were no subsequent problems, and the child was discharged 18 days after delivery. Case 4 was delivered vaginally at 39 weeks and 3 days, weighing 3,232 g with an Apgar Score of 9/9. Case 5 was delivered by cesarean section at another hospital; therefore, the details are unknown. All cases involved frozen embryos transferred during HRT cycles.

## Discussion

Frequency and causes of hypopituitarism

For female pituitary adenomas surgery and other treatments can significantly impair the patient’s fertility, requiring adequate counseling with the patient in advance. When hypopituitarism occurs and spontaneous conception becomes difficult, gonadotropins, GnRH pulse administration, and ART are necessary [3]. Four of the eight patients in this study had a history of pituitary adenoma surgery. All the patients in this study had hypopituitarism and required these drugs. Ascoli et al. reported the incidence of hypopituitarism as 12-42 cases per million women per year and a prevalence of 300-455 cases per million women per year; however, they underestimated the incidence, and the potential for hypopituitarism should have been considered when ART was performed [4]. Other studies have reported that GH deficiency can have detrimental effects on female reproductive functions, resulting in decreased ovarian reserve, ovarian response to COS, and embryo transfer success rates [5].

Pituitary function deficiency

Regarding ACTH, a study conducted on female horses demonstrated that abnormal ACTH blood levels strongly influence reproductive performance [6]. Therefore, unexplained infertility is a potential cause of pituitary hormone deficiency and should be considered high frequency in ART. Re-examining pituitary function in post-infection patients is necessary because COVID-19 may cause rapid fluctuations in pituitary function besides affecting the female and male reproductive organs [7].

Gonadotropin supplementation

Gonadotropins are hormones essential for reproduction. The FSH interacts with follicle receptors and is essential for mature follicles; LH interacts with follicle receptors and promotes follicle maturation, ovulation by LH surge, and progesterone production by the corpus luteum for fertilization and implantation.

In a systematic review of studies on human LH signaling, Armando reported that LH regulates the meiotic maturation of human oocytes, oocyte quality, and embryo quality and improves the quality of human oocytes and embryos [8]. We considered FSH and LH necessary for follicle development in hypopituitarism patients. We used hMG formulations containing LH or, in the case of rFSH formulations, weekly hCG administration as LH supplementation. As recombinant LH preparations have not been approved in Japan, we were unable to study the LH dosage. The results revealed that gonadotropin supplementation resulted in higher follicle growth and ovulation rates, consistent with previous reports. In addition, compared to the dosage in patients without hypopituitarism, the total dosage of the preparation needed to be higher because of the lack of endogenous gonadotropin secretion.

Regarding the daily dosage of FSH, the blood FSH levels were measured as a reference, and doses were adjusted based on the patient’s treatment history. Ovulation was achieved in the second cycle of treatment in two patients in whom the first cycle of treatment had failed.

Pituitary hormone effects

No uniform protocols exist for treating patients with pituitary insufficiency, and limited reports have been published on this topic. Scheffler et al. reported that GH is involved in ovarian follicle formation and oocyte maturation and that GH treatment improved oocyte and embryo quality in IVF [9]. Additionally, it reportedly stimulates responsiveness to ovarian stimulation and endometrial growth [10]. Milardi et al. also stated that GH deficiency is a major cause of reduced pregnancy rates and that in women, GH is a regulator of gonadotropins and has direct action on follicular maturation, in addition to mediating IGF-I, which amplifies both LH and FSH actions on the granule membrane [2,11].

Pregnancy

Reducing GH by 30%-50% is recommended after conception, as is increasing desmopressin according to clinical symptoms as adjustment of pituitary hormones after conception is necessary [12]. Pregnancy management during the gestational period is also important in individuals with established pregnancies; moreover, Verdú et al. reported that hypopituitarism did not affect pregnancy outcomes [13]; however, hypopituitarism was associated with an increased risk of perinatal complications, such as miscarriage, anemia, gestational hypertension, premature abruption of the common placenta, preterm delivery, and postpartum hemorrhage [1].

Vila et al. described pituitary hormone replacement during pregnancy, with glucocorticoids requiring a dose increase of 30%-40% in a small number of patients only in the third trimester, thyroid hormones requiring monitoring every four to six weeks, a reduction in free T4 GH by 30%-50% being recommended after conception, and an increase in desmopressin being recommended according to the clinical symptoms [11].

Although none of these complications occurred in the patients followed up at our hospital, their incidence is unknown because of the small number of cases. Therefore, management at a comprehensive perinatal center is desirable. Of the seven pregnancies achieved at our hospital, four (57%) resulted in miscarriages. Owing to the small number of cases, a causal relationship between pituitary hormone replacement and miscarriage is unknown. However, it should be considered.

As a limitation, this comparison was limited to the cases in which only rFSH was used. Another possibility is that the study was limited to a single institution, resulting in population limitations or biased stimulation methods. Retrospective methodology and number of cases are also limitations.

## Conclusions

The duration and total dose of rFSH administration are generally increased in hypopituitarism patients prior to egg retrieval, and egg retrieval efficiency is poor. On the other hand, pregnancy rates at the time of embryo transfer are as high as rates in patients without hypopituitarism. Further studies are required.
